# Editorial: Itch and Allergy

**DOI:** 10.3389/falgy.2021.804472

**Published:** 2021-11-25

**Authors:** Laurent Misery

**Affiliations:** ^1^University of Brest, LIEN, Brest, France; ^2^Department of Dermatology, University Hospital of Brest, Brest, France

**Keywords:** itch, mucosa, hymenoptera, contact dermatitis, autoimmunity, allergy, psychism

Itch (or pruritus) is an unpleasant condition leading to the need to scratch, as agonizing as pain (1). Since the 1990's, knowledges about itch are rapidly growing. More recently, an impressive number of new and future treatments is proposed to the medical community. It can be assumed that pruritus was initially a physiological reaction to signal the presence of insects on the surface of the skin and induce a scratching action to eliminate them. Due to the activation of processes involving neuro-immune interactions, pruritus also occurs during allergies. It can also occur during dermatological, neurological, psychiatric or multiple systematic diseases, and can also be iatrogenic. With the Research Topic Itch and allergy, we aim to increase awareness for this symptom and stimulate further basic, translational, clinical or psychological research in the field.

Cerpes et al. provided an interesting perspective on the role of Hymenoptera (especially sawflies, wasps, bees, and ants) which commonly induce itchy lesions. The complex process of venom activity and inflammation causes local reactions with pain and pruritus, sometimes anaphylactic reactions and more seldomly, as in case of numerous stings, systemic intoxication. They reviewed the literature regarding itch experienced after Hymenoptera stings, but surprisingly did not find any study that placed a specific focus on this topic. Peptide toxins from bee venom cause cell lysis and ion channel modulation in the peripheral and central nervous systems, while toxins from wasp venom induce mast cell degranulation and chemotaxis of polymorphonuclear leukocytes in the skin. Proteinaceous toxins cause a disruption of the cell membranes and necrotic cell death, degradation of hyaluronan (an extracellular matrix glycosaminoglycan), increased vascular permeability, hemolysis, as well as activated platelet aggregation. Mediators which could be directly involved in the venom-induced pruritus include histamine and tryptase released from mast cells, interleukin-4 and interleukin-13 from Th2 lymphocytes, as well as leukotriene C4.

Lambert brilliantly reviewed data on itch in allergic contact dermatitis. The immune response to contact allergy is very complex and not totally elucidated. Recently unique pathways preferentially activated by different allergens were identified. As for a lot of chronic itch disorders, antihistamines are ineffective for allergic contact dermatitis, suggesting a non-histaminergic itch. The exact mechanisms responsible for itch in these patients are still not well-defined. The most recent insights in pruritus in allergic contact dermatitis open perspectives for future therapies.

Lesslar and Smith called to mind that we traditionally think of itch as a sensation of the skin but that itching can also occur at mucosal sites and junctional dermal sites. They focused on specific itch patterns and discussed the pathophysiological mechanisms that underlie them. From an evolutionary point of view, these sites are guardian tissues as well as skin and are rich in sensory nerves and inflammatory cells. These authors overviewed the mechanisms by which itch may occur, how this may be amplified in specific tissue sites (nasal, ocular, auricular, oral, vulvovaginal, and anal itch), the clinical patterns this causes and some unique treatments.

Ferreira et al. tried to link pruritus, allergy and autoimmunity in an integrated model for a better understanding of psychodermatological interactions. Itch contributes greatly to the global and huge impact on quality of life related to skin disorders, both those which are not related to a primary dermatosis (illness) and those which are linked with primary skin lesions (disease). This is particularly evident within psychophysiological dermatoses, a group of psychodermatological diseases where there is a primary dermatosis, where psychological stress plays a role, and where pruritus may represent a major and shared symptom. The etiopathogenesis of pruritus in those disorders sheds light on the link among psychopathological features, psychological stress and the subtle interface between allergic and autoimmune mechanisms, where mast cells play a pivotal role. Biological mechanisms between allergy and autoimmunity, with key roles of mast cells and the connection with melatonin and immune-inflammatory pathways may be involved in the pathophysiology of psychophysiological dermatoses.

Hence, pruritus pathophysiology is not restricted to histamine. On the contrary, many neuroimmune interactions are involved and suggest very interesting perspectives for the management of allergic diseases.

## Author Contributions

The author confirms being the sole contributor of this work and has approved it for publication.

## Conflict of Interest

LM has received honoraria and/or research grants from the following companies: Abbvie, Almirall, Amgen, Bayer, Beiersdorf, Bioderma, Clarins, Estee Lauder, Expanscience, Galderma, Incyte, Johnson & Johnson, Kiniksa, L'Oréal, Leo, Lilly, Novartis, Pfizer, Pierre Fabre, Sanofi, Trevi, UCB, and Uriage.

## Publisher's Note

All claims expressed in this article are solely those of the authors and do not necessarily represent those of their affiliated organizations, or those of the publisher, the editors and the reviewers. Any product that may be evaluated in this article, or claim that may be made by its manufacturer, is not guaranteed or endorsed by the publisher.
